# Levels of ADAM10 are reduced in Alzheimer’s disease CSF

**DOI:** 10.1186/s12974-018-1255-9

**Published:** 2018-07-25

**Authors:** Aitana Sogorb-Esteve, María-Salud García-Ayllón, Johan Gobom, Jordi Alom, Henrik Zetterberg, Kaj Blennow, Javier Sáez-Valero

**Affiliations:** 1 Instituto de Neurociencias de Alicante, Universidad Miguel Hernández-CSIC, Av. Ramón y Cajal s/n, Sant Joan d’Alacant, E-03550 Alicante, Spain; 2 Centro de Investigación Biomédica en Red sobre Enfermedades Neurodegenerativas (CIBERNED), Sant Joan d’Alacant, Spain; 3 Unidad de Investigación, Hospital General Universitario de Elche, Fundación para el Fomento de la Investigación Sanitaria Biomédica de la Comunidad Valenciana (FISABIO), Elche, Spain; 4 Servicio de Neurología, Hospital General Universitario de Elche, Fundación para el Fomento de la Investigación Sanitaria Biomédica de la Comunidad Valenciana (FISABIO), Elche, Spain; 5 Clinical Neurochemistry Laboratory, Sahlgrenska University Hospital, Mölndal, Sweden; 6 Department of Neuroscience and Physiology, the Sahlgrenska Academy at the University of Gothenburg, Mölndal, Sweden; 7 Department of Molecular Neuroscience, UCL Institute of Neurology, Queen Square, London, UK; 8 UK Dementia Research Institute at UCL, London, UK

## Abstract

**Background:**

The disintegrin metalloproteinase 10 (ADAM10) is the main α-secretase acting in the non-amyloidogenic processing of the amyloid precursor protein. This study assesses whether ADAM10 is present in cerebrospinal fluid (CSF), and whether it has potential as a biomarker for Alzheimer’s disease (AD).

**Methods:**

ADAM10 was characterized in human CSF samples by immunoprecipitation and western blotting using antibodies specific for different domains of the protein and by ultracentrifugation in sucrose density gradients. Samples from AD patients (*n* = 20) and age-matched non-AD controls (*n* = 20) were characterized for classical CSF biomarkers, Aβ42, T-tau, or P-tau by ELISA, and assayed for soluble ADAM10 levels by western blotting.

**Results:**

We found that ADAM10 is present in human CSF as several distinct species: an immature form retaining the prodomain (proADAM10; ~ 80 kDa), a mature unprocessed full-length form (ADAM10f; ~ 55 kDa), and a truncated large soluble form released from the membrane (sADAM10; ~ 50 kDa). Fractionation by ultracentrifugation on sucrose density gradients showed that the ADAM10f and sADAM10 species form large complexes. Immunoblotting revealed a significant decrease in ADAM10f and sADAM10 in AD CSF compared to control CSF, while proADAM10 levels remained unaltered.

**Conclusions:**

Several forms of ADAM10 are present in CSF, mainly assembled as high-molecular weight complexes. The determination of the levels of mature forms of CSF-ADAM10 may be useful as a biomarker for AD.

**Electronic supplementary material:**

The online version of this article (10.1186/s12974-018-1255-9) contains supplementary material, which is available to authorized users.

## Background

The amyloid-β peptide (Aβ) is a key pathological effector of Alzheimer’s disease (AD) [1]. Aβ is a short polypeptide generated by processing of a larger type I transmembrane spanning glycoprotein, the amyloid precursor protein (APP), through the successive action of proteolytic enzymes called β-secretase and γ-secretase [2, 3]. APP can undergo alternative proteolytic processing [4]; indeed in the main pathway APP is cleavage by α-secretase within the Aβ domain, precluding Aβ formation [5]. Several members of membrane-bound disintegrin metalloproteinase (ADAM) family have been proposed as α-secretases, mainly ADAM10, ADAM17 (TACE), and ADAM9 [6], but other ADAM family members, such as ADAM8, may also cleave APP [7]. However, convincing evidence, particularly data from in vivo studies [8, 9], indicates that ADAM10 is the enzyme acting as the main physiologically relevant α-secretase [10].

The major neuronal β-secretase, the beta-site APP cleaving enzyme 1 (BACE1; [11] is present in CSF [12] in a soluble and truncated form, and increased β-secretase activity and BACE1 protein levels have been investigated as biomarkers for AD [13–16]. The presence in CSF of γ-secretase components, and particularly components of the catalytic subunit presenilin-1, have also been assessed recently as AD biomarkers [17, 18]. However, to our knowledge, only ADAM17/TACE activity has been assessed in both CSF [19] and plasma [20, 21]; while the potential of ADAM10 as an alternative AD biomarker has so far only been investigated in platelets [22, 23] and other blood cells [24]. ADAM proteases, similar to BACE1, are type I transmembrane proteins, but also include secreted isoforms [6]. Indeed, ADAM10 and ADAM17 have been shown to be secreted outside cells in exosomes [25]. Recently, an in-depth analysis of the human CSF endopeptidome enabled identification of several ADAM10 peptides [26].

In this study, we investigated the occurrence of ADAM10 in human CSF and whether altered levels of this protein occur in AD. We have characterized the full-length and truncated forms of ADAM10 in CSF, as well as immature forms of the protein that need to be taken into consideration for the design of an appropriate strategy for development of further assay approaches. We report that the full-length and truncated forms of ADAM10, but not the immature forms, decrease in AD CSF compared to control CSF.

## Methods

### Patients

CSF samples were obtained from the Clinical Neurochemistry Laboratory (Mölndal, Sweden) from patients who sought medical advice because of cognitive impairment. In total, 27 patients with AD (7 men and 20 women, mean age 71 ± 1 years) and 26 age-matched non-AD controls (NADC; 18 men and 8 women, mean age 70 ± 2 years) were included. Patients were designated as AD or NADC according to CSF biomarker levels using cutoffs that are > 90% specific for AD: total tau (T-tau) > 400 ng/L, P-tau > 60 ng/L and Aβ42 < 550 ng/L [27]. For details about classical CSF biomarker levels see Table 1. All AD patients fulfilled the 2011 NIA-AA criteria for dementia [28]. No other clinical data were available for the subjects. The CSF samples used for the present study were de-identified leftover aliquots from clinical routine analyses, following a procedure approved by the Ethics Committee at University of Gothenburg. This study was also approved by the Ethics Committee at the Miguel Hernandez University.

**Table 1:** Demographic data and classic CSF biomarker levels

Group	Age (years)	*n* (gender)	CSF Aβ42 (pg/mL)	CSF T-tau (pg/mL)	CSF P-tau (pg/mL)
NADC	70 ± 2 [55–88]	*n* = 26 (8F/18M)	773 ± 29 [1010–561]	238 ± 13 [138–365]	36 ± 2 [21–51]
AD	71 ± 1 [55–86]	*n* = 27 (20F/7M)	414 ± 15* [544–251]	689 ± 48* [1420–443]	88 ± 5* [164–61]

Patients were designated as NADC or AD according to CSF biomarker levels using cutoffs as described in the “Methods” section (and ref. [27]). The data represent the means ± SEM. The ranges of values for each variable are also indicated. *F* female; *M* male. *Significantly different (*p* < 0.001) from the NADC group

### Cell cultures

For obtaining conditioned cell-culture medium CHO cells (450,000 cells/well) were grown in six-well plates for 48 h in Dulbecco’s modified Eagle’s medium (DMEM) plus GlutaMAX™ (Gibco® Life Technologies, Paisley, UK) supplemented with 5, 1 or 0.5% fetal bovine serum (FBS; Gibco) and 100 μg/mL penicillin/streptomycin (Gibco). After 48 h, the cell medium was recollected, centrifuged for 15 min at 1500×*g* at 4 °C, and frozen for future analysis.

### Western blotting and biochemical measurements

Samples of CSF (30 μL) and cell medium (20 μL) were denatured at 98 °C for 5 min and resolved by sodium dodecyl sulfate-polyacrylamide gel electrophoresis (SDS-PAGE) under reducing conditions. Following electrophoresis, proteins were blotted onto nitrocellulose membranes (Bio-Rad Laboratories GmbH, Munich, Germany). Bands of ADAM10 immunoreactivity were detected using an antibody specific for the mid-region of ADAM10 (hereafter referred to as the ectodomain antibody; rabbit polyclonal; OAGA02442, Aviva Systems Biology, San Diego, USA), an anti-N-terminal (rabbit polyclonal, ab39153, Abcam, Cambridge, UK), and anti-C-terminal ADAM10 antibody (rabbit monoclonal; ab124695, Abcam). Blots were then probed with the appropriate conjugated secondary antibodies, and imaged on an Odyssey Clx Infrared Imaging System (LI-COR Biosciences, Lincoln, NE, USA). Band intensities were analyzed using LI-COR software (Image Studio Lite). All samples were analyzed at least in duplicate. Ponceau staining served to monitor potential loading inaccuracies in individual blots. Immunoreactive ADAM10 signal for each band was normalized to the immunoreactivity of the corresponding band from a control CSF sample (aliquots from the same sample), resolved in all the blots. For the estimation of the (50 + 55 kDa)/80 kDa ratio for each sample (see the “Results” section), the unprocessed immunoreactivity for each band was considered.

Serum and CSF albumin concentrations were measured by immunonephelometry on a Beckman Image Immunochemistry system (Beckman Instruments, Beckman Coulter). The CSF/serum albumin ratio was calculated as CSF albumin (mg/L)/serum albumin (g/L). CSF albumin levels were also estimated by western blotting using a rabbit polyclonal antibody (SAB2100098, Sigma-Aldrich, Saint Louis, USA).

### Sucrose density gradient ultracentrifugation

ADAM10 complexes were fractioned by ultracentrifugation at 250,000×*g* on a continuous sucrose density gradient (5–20%) for 4 h at 4 °C in a Beckman TLS 55 rotor. CSF aliquots (65 μL) were carefully loaded onto the top of the gradient containing 2 mL of 0.15 M NaCl, 50 mM MgCl_2_, and 0.5% Brij 97 in 50 mM Tris-HCl (pH 7.4). After centrifugation, ~ 14 fractions were collected gently from the top of the tubes. Enzyme markers of known sedimentation coefficient, β-galactosidase, catalase, and alkaline phosphatase were used in the gradients to determine the approximate sedimentation coefficients.

### Measurement of T-tau, P-tau, and Aβ42 by ELISA

Total tau (T-tau), phosphorylated tau (P-tau), and Aβ1–42 (Aβ42) concentrations in CSF were measured using INNOTEST ELISA methods (Fujirebio Europe, Gent, Belgium).

### Statistical analysis

All the data were analyzed using SigmaStat (Version 3.5; Systac Software Inc.) using a Student’s *t* test (two-tailed) or a Mann-Whitney *U* test for single pairwise comparisons, and determining the exact *p* values. The results are presented as means ± SEM, and the correlation between variables was assessed by linear regression analyses.

## Results

### ADAM10 is present in human CSF as several species

**Fig. 1:**
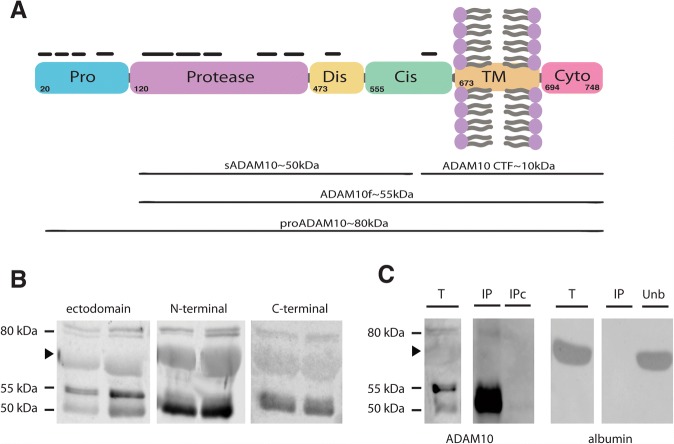
Different ADAM10 species are present in human CSF. **a** Schematic representation of ADAM10 and its domain organization, which consists of a pro-domain (Pro), a zinc-binding metalloprotease (Protease) domain, a disintegrin domain (Dis), which binds to integrin cell adhesion molecules, a cysteine-rich domain (Cys), a variable stalk region, a transmembrane (TM) domain, and a cytosolic domain (Cyto). Not drawn in scale; adapted from [29]. The potential species resulting from proteolytic removal of the prodomain that are further released from the membrane are indicated (immature form, proADAM10; mature full-length form, ADAM10f; truncated soluble form, sADAM10). The approximate localizations matching identified ADAM10 peptides in human CSF by nano-LC-MS analysis are indicated (see Additional file 1: TableS1 for additional details). **b** Western blot of human CSF samples from controls (non-AD) subjects, resolved with the indicated anti-ADAM10 antibodies. Arrow head indicates a non-specific band (see C). **c** Control CSF samples were immunoprecipitated with the ADAM10 N-terminal antibody, and precipitated proteins (IP) were immunoblotted with either ADAM10 (ectodomain) or albumin antibodies. The same CSF samples were incubated, in parallel, with a non-specific rabbit IgG and analyzed as negative controls (IP control, IPc). For the blot resolved for albumin, the unbound fraction (Unb) is also shown to demonstrated that ADAM10 antibodies is not able to pull-down albumin

ADAM10 is expressed as a 748 amino-acid-residue type I glycoprotein composed of an N-terminal signal sequence followed by a prodomain, a metalloprotease domain, a disintegrin domain, a cysteine-rich region, a transmembrane helix, and a cytoplasmic region (Fig. 1a). In a previous in-depth LC-MS analysis of human CSF peptides, we were able to identify 38 small peptides matching parts of the ADAM10 sequence, including the prodomain and the cysteine-rich region close to the transmembrane domain [26] (see also Fig. 1a and Additional file 1: TableS1). Analysis of control CSF samples by SDS-PAGE and western blotting using ectodomain and N-terminal ADAM10 antibodies revealed three immunoreactive species with apparent molecular masses of ~ 80, 55, and 50 kDa (Fig. 1b), and a broad band of ~ 70 kDa (expected molecular mass of albumin). Immunoblotting with an anti-C-terminal ADAM10 antibody detected the 80 and 55 kDa, as well a broad 70 kDa band, but not a 50 kDa band (Fig. 1b), suggesting that the 50 kDa form of CSF ADAM10 is C-terminally truncated. To further examine the identity of ADAM10 immunoreactive species in human CSF, we performed immunoprecipitation/western blot analysis (Fig. 1b). CSF samples were immunoprecipitated using the anti-N-terminal ADAM10 antibody, and blots stained with the ectodomain antibody detecting the 80 kDa species and a strongly immunoreactive 50–55 kDa band. These bands were not observed in negative immunoprecipitation controls, including when an irrelevant rabbit IgG was used (Fig. 1c). The ~ 70 kDa band was not present in ADAM10 immunoprecipitates of CSF. When blots were stained with an anti-albumin antibody a 70 kDa band was observed in the unbound fraction (Fig. 1c). We did not observe immunoreactivity when blots were resolved with a control lacking primary antibody (blots not shown).

Accordingly, based on the pattern of immunoreactivity with the different antibodies, and the apparent molecular mass of ADAM10 species reported previously [10, 22, 29, 30], we attributed the 80 kDa band to the immature form of ADAM10 (proADAM10), and the 55 kDa form to the mature form (full-length, ADAM10f) derived from the proADAM10 form by removal of the prodomain (194 aa, [31, 32]), and the 50 kDa form to a truncated ADAM10 (soluble, sADAM10), released from the membrane by metalloproteases (ADAM9/15; [29]).

Interestingly, immunoblotting of CHO cell-conditioned medium with an anti-C-terminal ADAM10 antibody revealed the presence of bands attributed to soluble ADAM10f and proADAM10 species (Additional file 2: FigureS1a). The ~ 70 kDa band was also observed in cell-conditioned medium, but the immunoreactivity of the band declined as the amount of FBS in the culture medium was lowered (Additional file 2: FigureS1b), confirming that this band may represent nonspecific staining associated to albumin.

### ADAM10 species in CSF form complexes

**Fig. 2:**
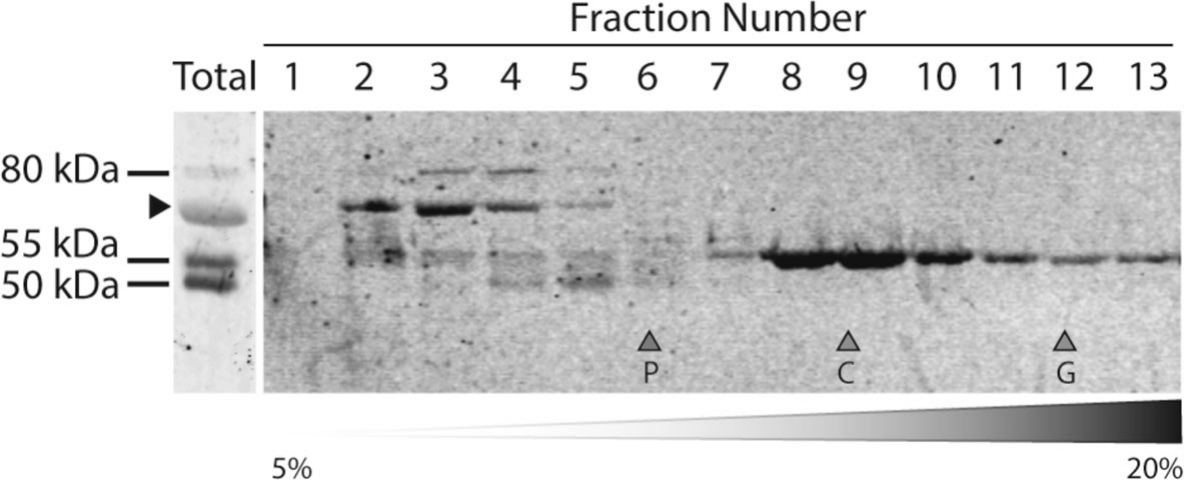
Characterization of CSF-ADAM10 complexes by sucrose gradient ultracentrifugation. CSF samples (NADC) were fractionated on 5–20% sucrose density gradients. The fractions (collected from the top of each tube) were immunoblotted using the ectodomain antibody specific for a domain common to all the soluble CSF-ADAM10 species. Enzymes of known sedimentation coefficient, alkaline phosphatase (P, 6.1S; ~ 140–160 kDa), catalase (C, 11.4S; ~ 232 kDa), and β-galactosidase (G, 16.0S; ~ 540 kDa) were used as internal markers. A representative case from three independent experiments is shown

Since ADAM10 exists at the plasma membrane as dimers [33], we characterized the occurrence of CSF-ADAM10 oligomers by gradient ultracentrifugation, which has previously served to illustrate the existence of different protein complexes in CSF [34, 35]. Western blotting under denaturing conditions using the ADAM10 ectodomain antibody, common to all species, showed that the proADAM10 species accumulated before to the alkaline phosphatase marker (molecular mass ~ 140–160 kDa), while the sADAM10 species were identified in denser fractions, between the alkaline phosphatase and catalase (molecular mass ~ 232 kDa). Interestingly, the ADAM10f species were resolved in the denser fractions, close to β-galactosidase (molecular mass ~ 540 kDa) (Fig. 2). The particular sedimentation pattern for each ADAM10 species indicated that, at least, sADAM10 and ADAM10f could form large complexes in CSF.

### Mature forms of ADAM10 are decreased in AD CSF

**Fig. 3:**
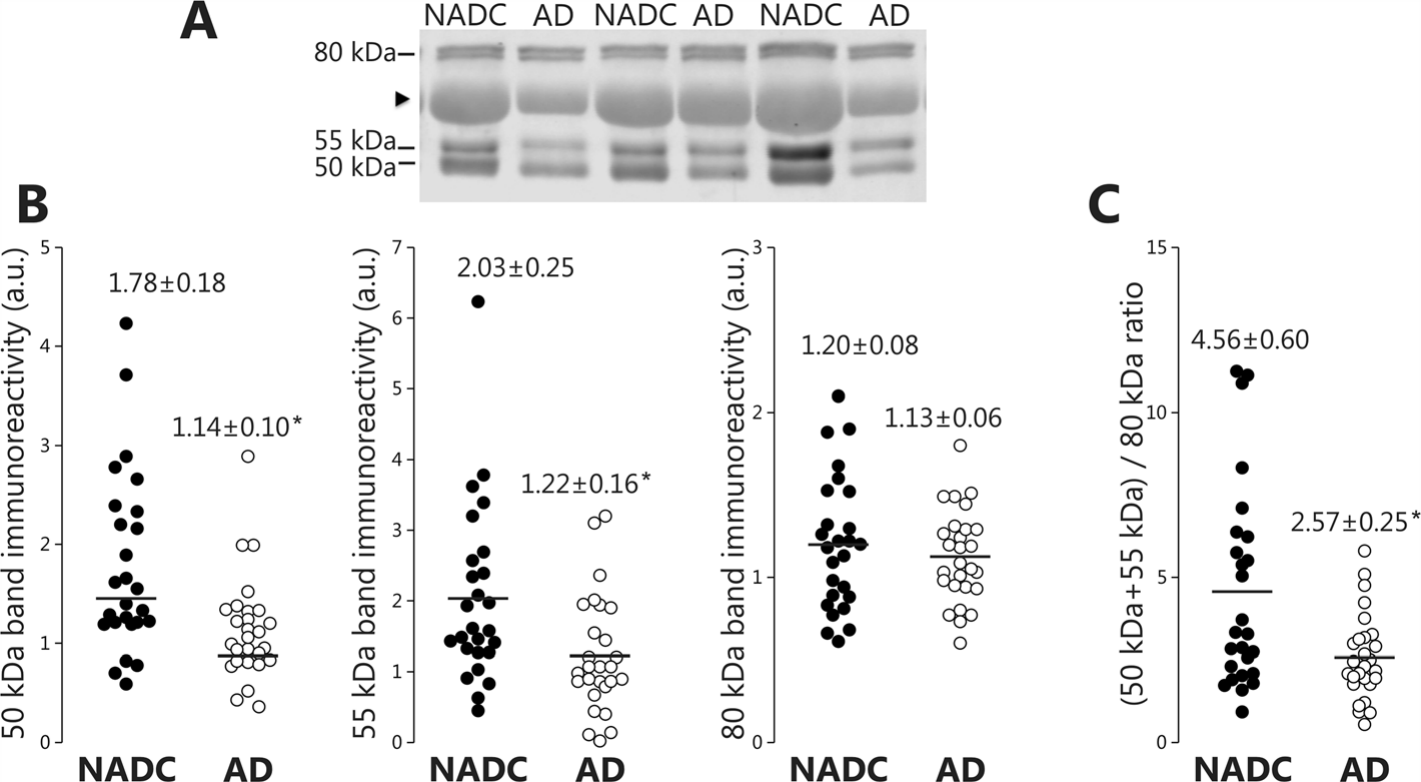
Decreased levels of mature ADAM10 species in AD CSF samples. **a** Representative blot of ADAM10 species immunoreactive to an ectodomain antibody in the CSF samples from 27 AD patients and 26 age-matched non-AD controls (NADC). Arrow head indicates a non-specific band. **b** Densitometric quantification of ADAM10 immunoreactivity from the 55 kDa species attributed to the mature form (ADAM10f), the truncated 50 kDa (sADAM10), and the 80 kDa immature form (proADAM10). Equal volumes of CSF were loaded in each lane and Ponceau staining served to monitor potential loading inaccuracies (none of the major bands detected by Ponceau staining displayed significant differences between groups). **c** The ratio derived from the immunoreactivity for the mature 50 and 55 kDa bands relative to that for the immature 80 kDa band estimated in each sample [(50 kDa + 55 kDa)/80 kDa] is also shown. Data are presented as means ± SEM: *p ≤ 0.005

After assigning the different ADAM10 immunoreactive species present in CSF as full-length (~ 55 kDa, ADAM10f) or truncated (~ 50 kDa, sADAM10) mature species, as well as immature forms (~ 80 kDa, proADAM10), we assessed whether the concentrations of these species are altered in AD. We analyzed CSF samples from 27 AD patients and 26 NADC. The core AD biomarkers were measured using ELISA, confirming elevated CSF T-tau and P-tau and low levels of Aβ42 in the AD samples (Table 1). Regarding ADAM10 immunoreactivities, we found that the 55 kDa species decreased in abundance (~ 40%; *p* = 0.005) in AD compared to NADC subjects (Fig. 3). A similar decrease was found for the truncated 50 kDa fragment (~ 36%; *p* = 0.004), whereas, the concentration of the 80 kDa immature ADAM10 form was unchanged (*p* = 0.44). Despite the different evolution of the mature and immature ADAM10 species, we observed a trend towards a decrease in the non-specific 70 kDa band, attributed to albumin. The CSF/serum albumin ratio is accepted as a more sensitive and adequate parameter for the demonstration of a blood-brain damage than CSF-protein or CSF-albumin [36]. We recently studied the CSF/serum albumin ratio in a large cohort of patients diagnosed with AD and found no evidence of blood-brain barrier dysfunction in AD. We assume that a higher CSF/serum albumin may represent concomitant cerebrovascular pathology [37]. In the patient cohort included in this study, we found a non-significant (*p* = 0.063) trend towards a decrease in the CSF/serum albumin ratio for the AD subjects (5.8 ± 0.6) compared to NADC (7.4 ± 0.6). For this reason, to prevent a potential misinterpretation of the decrease in ADAM10 species, we examined the (50 + 55 kDa)/80 kDa ratio for each sample based on the direct analysis of the western blots. Accordingly to our previous analysis, the ratio of mature/immature ADAM10 species also displayed differences between AD and NADC groups, indicating that a decrease in mature ADAM10 species in AD is not associated with a lower protein content of the AD samples or a loading artifact (Fig. 3c).

We further tested whether CSF-ADAM10 complexes are altered in AD cases. CSF-ADAM10 complexes were fractioned, from three AD and three NADC representative cases, using sucrose-density gradient fractionation, and then resolved by western blotting under denaturing conditions (Additional file 3: FigureS2). Peaks of the CSF-ADAM10 complexes were identified in similar fractions for all the AD and ND cases tested, indicating that all complexes are present in AD CSF, and the nature of the complexes is not affected by the pathological conditions.

Interestingly, levels of sADAM10 and ADAM10f were correlated, albeit weakly, in CSF from NADC subjects (*R* = 0.39; *p* = 0.048), but not in AD patients (*R* = 0.25; *p* = 0.20). In NADC subjects, no correlation was observed between proADAM10 and ADAM10f (*R* = 0.30; *p* = 0.13) or proADAM10 and sADAM10 (*R* = 0.15 *p* = 0.46). In the AD subjects, these correlations were not evident for both proADAM10 and ADAM10f (*R* = 0.05; *p* = 0.81) or for proADAM10 and sADAM10 (*R* = 0.12; *p* = 0.56). No correlations were observed with age. Although the groups were not balanced regarding gender (see Table 1), we did not observe differences in any parameter between males and females in the AD group. Levels of the ADAM10 species did not correlate with the core AD biomarkers in NADC, while in AD samples, higher Aβ42 levels correlated with lower ADAM10f levels (*R* = 0.43; *p* = 0.027), but not correlations were observed with the levels of sADAM10 species (Additional file 4: FigureS3).

## Discussion

There is a need to identify additional CSF biomarkers of AD. The knowledge that APP metabolism and Aβ production and aggregation are key steps in AD pathogenesis makes proteins involved in the pathological processing of APP, including secretases such as ADAM10, reasonable candidates for analysis in CSF. However, since secretases are transmembrane proteins, their assessment in CSF was not considered until recent years.

Previous studies have revealed that, in addition to proteins, CSF contains many endogenous peptides [38, 39], including ADAM10 peptides [26]. In this study, we demonstrate the presence in human CSF of the mature and immature full-length ADAM10 protein, as well as a membrane cleaved large fragment (sADAM10). As sADAM10 can be released by proteolytic processing from the membrane [29], this suggests the potential for truncated isoforms to be present in CSF. Indeed, recent reports indicate the possibility that ADAM10 levels can even be measured in human serum by an enzyme-linked immunosorbent assay (ELISA; [40, 41].

In our previous study [26] using LC-MS analysis, we identified several short peptide fragments of ADAM10 in human CSF, matching sequences located at the N-terminus of the protein as well peptide fragments located close to the transmembrane domain of the protein. In this study, several different molecular mass bands of ADAM10 were detected by western blot analysis using N- and C-terminal anti-ADAM10 antibodies. Thus, in addition to a sADAM10 isoform attributed to the immunoreactive band of ~ 50 kDa molecular mass, other ADAM10 species retaining the intracellular C-terminal domain are present in the CSF. Moreover, as some of the sequences identified by LC-MS analysis were homologous to the N-terminal prodomain, this indicated that, unexpectedly, immature proADAM10 also reached the CSF. Thus, other full-length isoforms of the protein co-exist in the CSF with sADAM10. The presence of proADAM10, together with ADAM10f, has been described at the cell surface [10].

The mechanisms by which these membrane-resident ADAM10 species reach the CSF are unknown, but neuronal death may be a contributing factor. Interestingly, proADAM10 and ADAM10f were also detected in culture media from CHO cells. Abundant ADAM10 has been found in exosomes of bovine endometrial stromal cells cultured at hypoxic conditions [42]. Thus, an exosomal contribution of ADAM10-CSF cannot be discounted. Interestingly, ADAM10 is also enriched in synaptic vesicles [43], being one of many synaptic proteins identified and measured in CSF [44]. In this context, we and others have reported evidence of the presence in the CSF of “unprocessed” forms of several transmembrane proteins, such as BACE1 [34], APP [35, 45], and the multi-pass presenilin-1 (PS1) [18, 34]. Thus, the existence of a membrane-resident protein in CSF is not an unusual finding [46]. Recently, we also characterized in CSF the existence of C-terminal fragments of APP, which include the transmembrane domain [47].

The occurrence in CSF of proteins which still maintain their transmembrane and intracellular domains is also relevant for the development of strategies for their quantitative estimation. ADAM10, similar to many other transmembrane proteins exists as a dimer in the brain [33]. Both the transmembrane [48] and cytoplasmic [33] domains can participate in dimerization of ADAM10, a feature that may be is an inherent property of ADAM metalloproteinases. In the present study, we demonstrated by gradient centrifugation that sADAM10 and ADAM10f are present in the CSF as large complexes. Further studies will be necessary to clarify the biochemical properties of these homomeric complexes, but our preliminary analysis indicates that the species in NADC CSF are similar, if not identical to the species in the AD CSF. We have previously demonstrated the occurrence of APP heteromers in CSF, comprising both sAPPα/sAPPβ and also soluble full-length APP, and we have shown that these heteromers affect the determination of sAPP by ELISA [35]. Given that the distinct ADAM10 species also form complexes, the development of an accurate ELISA protocol for the estimation of CSF-ADAM10 levels may require more knowledge about the potential variable stoichiometry and stability of these complexes. In fact, our early attempts to assess ADAM10-CSF levels by ELISA have resulted in poorly reproducible data (~ 60% intra-assay variability in CSF samples; ELISA kit from MyBioSource, Inc. San Diego, CA, USA). A previous study also reported difficulties in assessing ADAM10 in CSF, discarding their presence in the fluid [49]. In this study, to circumvent this issue, we analyzed ADAM10-CSF levels by SDS-PAGE.

Our determination of the different species of ADAM10 in CSF by western blotting indicated that, in AD cases, there is a decrease in sADAM10 and ADAM10f, but not in the immature forms. Since amyloidogenic processing of APP is expected to be altered in the Alzheimer brain, parallel changes in the levels of α-secretase and β-secretase might be expected. However, it is still unclear if α-secretase and β-secretase are inversely correlated during pathological progression, as the proteolytic products sAPPα and sAPPβ displayed similar trends in the CSF [45]. Data on ADAM10 in human brain are scarce, but the majority of the data indicate an overall decrease in ADAM10 mRNA, protein, and/or activity in the brain of AD patients compared to age-matched controls [50]. However, at least in platelets, the decrease of ADAM10 protein in AD patients is not caused by a reduction in ADAM10 mRNA [51]. Thus, the regulation of expression and activity of ADAM10 may be complex, being regulated by several pathways, epigenetically, and at translational and post-translational levels [50], and affected by normal aging [30]. In this context, it may be important to evaluate α-secretase activity in CSF. Enzymatic activity assays in CSF are usually based on the use of specific substrates (synthetic peptides) favorable for the assessment of a concrete activity, but as mentioned previously, other enzymes, in addition to ADAM10, display α-secretase-like activity. Indeed, elevated activity levels for ADAM17/TACE have been found in both CSF [19] and plasma [20, 21] from subjects with AD. It appears important to decipher the physiopathological significance of the differential regulation in AD for ADAM10 and ADAM17. Anyhow, in the view of the apparent increase in ADAM17 levels and decrease in ADAM10 protein levels in AD CSF, it is questionable whether an enzymatic activity assay for ADAM10 in CSF, based in the use of synthetic peptides (which can be cleaved by multiple proteases), should be used to measure changes in the CSF α-secretase activity of AD patients. The general requirements for secretase cleavage are not strict, and we cannot exclude the possibility that other CSF enzymes that may cleave the synthetic peptides also being detected. Moreover, emerging evidence indicates that the plasma membrane with its unique dynamic properties may additionally play an important role in controlling sheddase function, as physicochemical properties of the lipid bilayer that govern the action of ADAM-proteases [52]. Accordingly, determination of enzymatic activities does not appear to be the most adequate and sensitive molecular tool to evaluate ADAM10, and other secretases, as a potential CSF biomarkers. Therefore, only ELISA assays based on pan-specific antibodies for concrete ADAM10 species, and including pre-treatment methods designed to disaggregate complexes, may be a reliable approach to assess ADAM10 protein levels and enzymatic activity.

Despite the limited precision of western blotting for quantitative analysis, we consider that mature forms of ADAM10 in CSF constitute potential new biomarkers of AD. The western blotting technique is also an important limitation regarding the number of cases to analyze. A corroborative study in a large and independent cohort should be necessary to assess the potential of this new biomarker for AD diagnosis. New studies should also address analysis of aging in normal conditions, adequate balance of gender in the pathological group, and more importantly, the specificity of the changes for AD confronting other dementias. In this study, we defined neurochemically AD and NADC subjects based on the levels of classical AD biomarkers. The NADC subjects included in this study also exhibited cognitive impairment; therefore, the control group is not totally comprised of healthy individuals and probably includes several non-AD conditions. Despite the inherent difficulty of the clinical diagnosis to truly assess the potential of new diagnostic biomarkers, tentative studies including other dementias clinically diagnosed will be of particular relevance, especially with longitudinal follow-up and establishment of definitive diagnosis. The involvement of ADAM10 in other pathological processes such as traumatic brain injury, inflammation, brain tumors, stroke, and psychiatric diseases [48, 53] also deserves the analysis of CSF-ADAM10 levels.

## Conclusions

Our present findings provide sufficient evidence to justify further studies focusing on the possibility of monitoring specific soluble forms of ADAM10 and to evaluate the progress and feasibility of developing molecular tools for this potential new CSF biomarker for AD.

## Additional files

Additional file 1: **Table S1.** Identified ADAM10 peptides in CSF. (DOCX 17 kb)

Additional file 2: **Figure S1.** Soluble mature and immature ADAM10 species are present in cell medium by CHO cells. **a** Western blot of cell extract (cell) and culture medium (cell media) from CHO cell cultures grown in presence of 5% FBS, resolved with the anti-ADAM10 C-terminal antibody. b Cell medium from CHO cells grown in presence of 0.5 or 1% FBS are also shown. Arrow head indicates a non-specific band attributed to albumin (see Fig. 1). (TIF 6444 kb)

Additional file 3: **Figure S2.** Unaltered ADAM10 complexes in AD CSF. (A) Representative blot of ADAM10 complexes in CSF from AD subjects and age-matched non-AD controls (NADC). Three representative AD and NADC cases were analyzed, in which the distribution of ADAM10 complexes displayed similar sedimentation patterns. Blots were resolved with an ADAM10 ectodomain antibody (domain common to all the CSF-ADAM10 species). (TIF 1707 kb)

Additional file 4: **Figure S3.** Correlation of mature ADAM10 species with Aβ42 levels in CSF samples. A linear regression analysis was used to assess the correlation between the Aβ42 levels obtained by ELISA (see Table 1) and ADAM10f, or sADAM10 in the samples from age-matched NADC (closed symbol, solid lines) and AD patients (open symbol, dotted lines). Correlations for T-tau or P-tau were non-significant (not shown). The linear regression coefficient (R) and *p* values for each correlation are shown (n.s.: non-significant *p* value). (TIF 476 kb)

## Abbreviations

AD: Alzheimer’s disease
ADAM10: Disintegrin metalloproteinase 10
ADAM10f: Full-length form of ADAM10
APP: Amyloid precursor protein
Aβ: Amyloid-β peptide
BACE1: Beta-site APP cleaving enzyme 1
CSF: Cerebrospinal fluid
DMEM: Dulbecco’s modified Eagle’s medium
NADC: Non-AD controls
proADAM10: Prodomain of ADAM10
PS1: Presenilin-1
P-tau: Phosphorylated tau
sADAM10: Soluble form of ADAM10 released from the membrane
SDS-PAGE: Sodium dodecyl sulfate-polyacrylamide gel electrophoresis
T-tau: Total tau
