# Supplementary material for: Levels of ADAM10 are reduced in Alzheimer’s disease CSF
Source: J Neuroinflammation. 2018 Jul 25;15:213. doi: 10.1186/s12974-018-1255-9 (PMC6060469; doi:10.1186/s12974-018-1255-9)
Supplement: Supplementary file 1 — Table S1. Identified ADAM10 peptides in CSF. (DOCX 17 kb) [file 12974_2018_1255_MOESM1_ESM.docx]

**Table S1.** Identified ADAM10 peptides in CSF.

| **Positions in protein** | **Amino acid sequence** | **Sequence length** | **Theo. MH+ [Da]** |
| --- | --- | --- | --- |
| 132-142 | R.GGTFYVEPAER.Y | 11 | 1224,58 |
| 132-147 | [R].GGTFYVEPAERYIKDR.[T] | 16 | 1900,96 |
| 155-177 | V.IYHEDDINYPHKYGPQGGCADHS.V | 23 | 2672,14 |
| 167-181 | [K].YGPQGGCADHSVFER.[M] | 15 | 1679,72 |
| 167-183 | K.YGPQGGCADHSVFERMR.K | 17 | 1965,86 |
| 184-207 | [R].KYQMTGVEEVTQIPQEEHAANGPE.[L] | 24 | 2685,25 |
| 187-207 | [Q].MTGVEEVTQIPQEEHAANGPE.[L] | 21 | 2266,03 |
| 188-202 | [M].TGVEEVTQIPQEEHA.[A] | 15 | 1666,79 |
| 188-203 | [M].TGVEEVTQIPQEEHAA.[N] | 16 | 1737,83 |
| 188-206 | [M].TGVEEVTQIPQEEHAANGP.[E] | 19 | 2005,95 |
| 188-207 | M.TGVEEVTQIPQEEHAANGPE.L | 20 | 2133,98 |
| 189-207 | [T].GVEEVTQIPQEEHAANGPE.[L] | 19 | 2033,94 |
| 190-208 | [G].VEEVTQIPQEEHAANGPEL.[L] | 19 | 2090,00 |
| 190-209 | [G].VEEVTQIPQEEHAANGPELL.[R] | 20 | 2203,09 |
| 19-29 | G.GQYGNPLNKYI.R | 11 | 1265,64 |
| 193-207 | [E].VTQIPQEEHAANGPE.[L] | 15 | 1619,77 |
| 200-209 | [E].EHAANGPELL.[R] | 10 | 1050,52 |
| 214-231 | R.TTSAEKNTCQLYIQTDHL.F | 18 | 2122,00 |
| 32-43 | [H].YEGLSYNVDSLH.[Q] | 12 | 1396,64 |
| 32-47 | [H].YEGLSYNVDSLHQKHQ.[R] | 16 | 1917,91 |
| 33-43 | [Y].EGLSYNVDSLH.[Q] | 11 | 1233,57 |
| 33-44 | [Y].EGLSYNVDSLHQ.[K] | 12 | 1361,63 |
| 33-45 | [Y].EGLSYNVDSLHQK.[H] | 13 | 1489,73 |
| 33-47 | [Y].EGLSYNVDSLHQKHQ.[R] | 15 | 1754,85 |
| 33-49 | [Y].EGLSYNVDSLHQKHQRA.[K] | 17 | 1981,98 |
| 34-47 | E.GLSYNVDSLHQKHQ.R | 14 | 1624,80 |
| 386-406 | [V].GHNFGSPHDSGTECTPGESKN.[L] | 21 | 2214,91 |
| 388-408 | H.NFGSPHDSGTECTPGESKNLG.Q | 21 | 2189,93 |
| 389-406 | [N].FGSPHDSGTECTPGESKN.[L] | 18 | 1906,79 |
| 421-436 | R.ATSGDKLNNNKFSLCS.I | 16 | 1754,83 |
| 502-518 | [K].QCSPSQGPCCTAQCAFK.[S] | 17 | 1986,79 |
| 52-63 | [R].AVSHEDQFLRLD.[F] | 12 | 1429,71 |
| 54-63 | [V].SHEDQFLRLD.[F] | 10 | 1259,60 |
| 55-63 | S.HEDQFLRLD.F | 9 | 1171,56 |
| 639-645 | [Y].CDVFMRC.[R] | 7 | 987,38 |
| 78-91 | [R].DTSLFSDEFKVETS.[N] | 14 | 1604,73 |
| 83-96 | [F].SDEFKVETSNKVLD.[Y] | 14 | 1610,79 |
| 84-94 | S.DEFKVETSNKV.L | 11 | 1294,64 |
