# Supplementary figures and images for: Levels of ADAM10 are reduced in Alzheimer’s disease CSF
Source: J Neuroinflammation. 2018 Jul 25;15:213. doi: 10.1186/s12974-018-1255-9 (PMC6060469; doi:10.1186/s12974-018-1255-9)

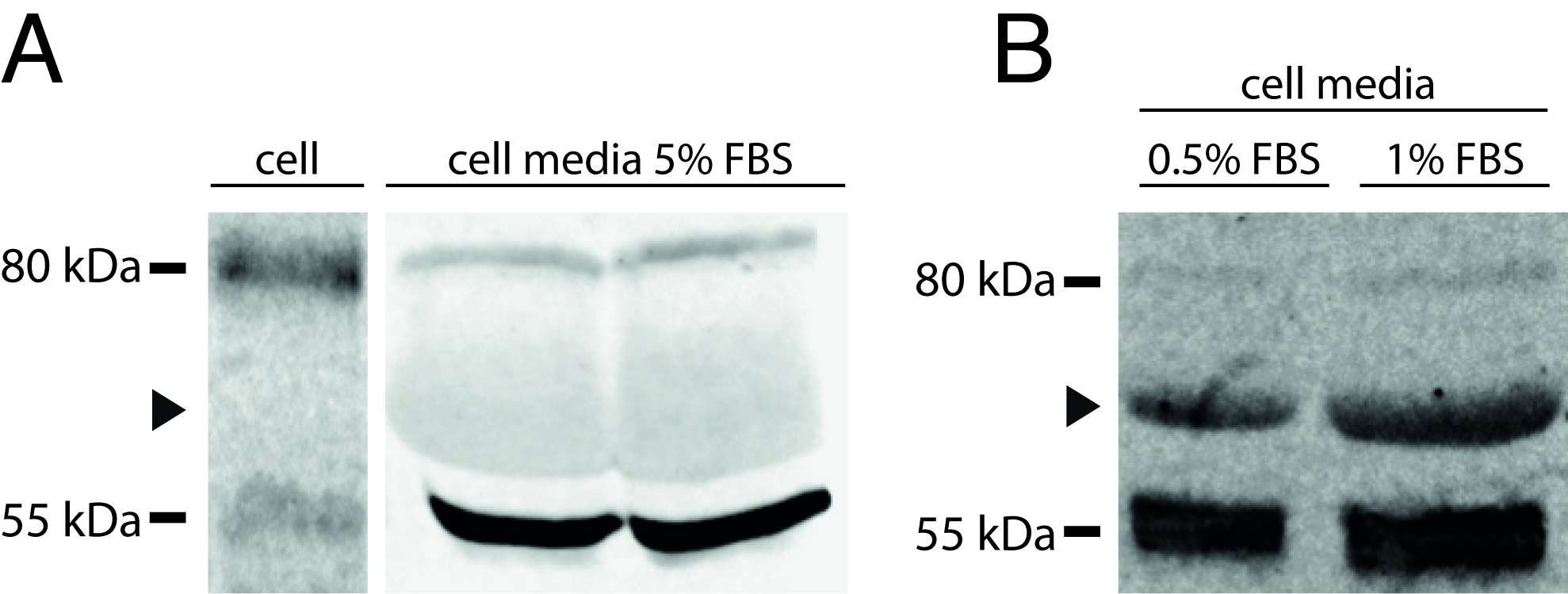

Supplement: Supplementary file 2 — Figure S1. Soluble mature and immature ADAM10 species are present in cell medium by CHO cells. a Western blot of cell extract (cell) and culture medium (cell media) from CHO cell cultures grown in presence of 5% FBS, resolved with the anti-ADAM10 C-terminal antibody. b Cell medium from CHO cells grown in presence of 0.5 or 1% FBS are also shown. Arrow head indicates a non-specific band attributed to albumin (see Fig. 1). (TIF 6444 kb) [file 12974_2018_1255_MOESM2_ESM.tif]

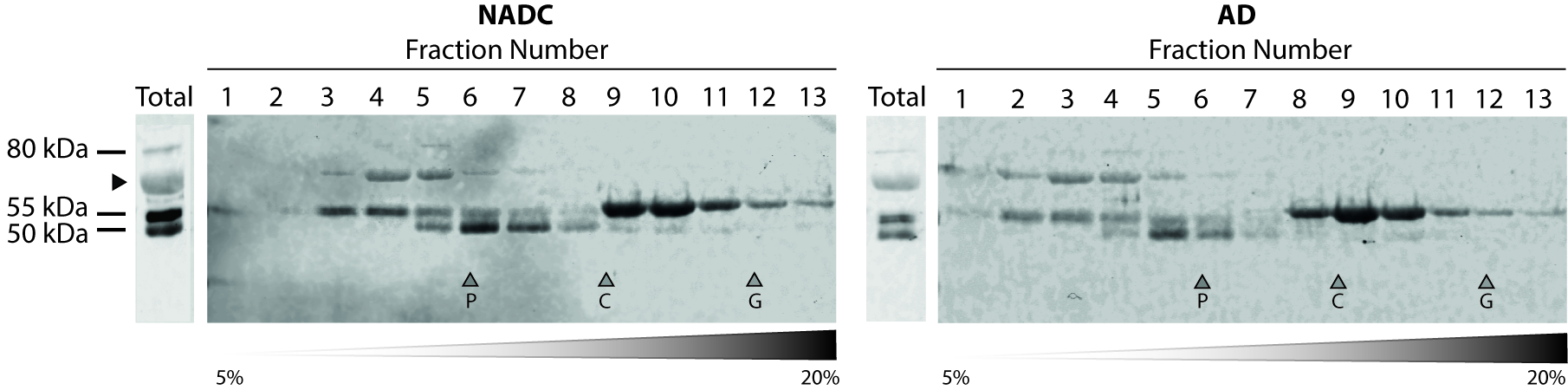

Supplement: Supplementary file 3 — Figure S2. Unaltered ADAM10 complexes in AD CSF. (A) Representative blot of ADAM10 complexes in CSF from AD subjects and age-matched non-AD controls (NADC). Three representative AD and NADC cases were analyzed, in which the distribution of ADAM10 complexes displayed similar sedimentation patterns. Blots were resolved with an ADAM10 ectodomain antibody (domain common to all the CSF-ADAM10 species). (TIF 1707 kb) [file 12974_2018_1255_MOESM3_ESM.tif]

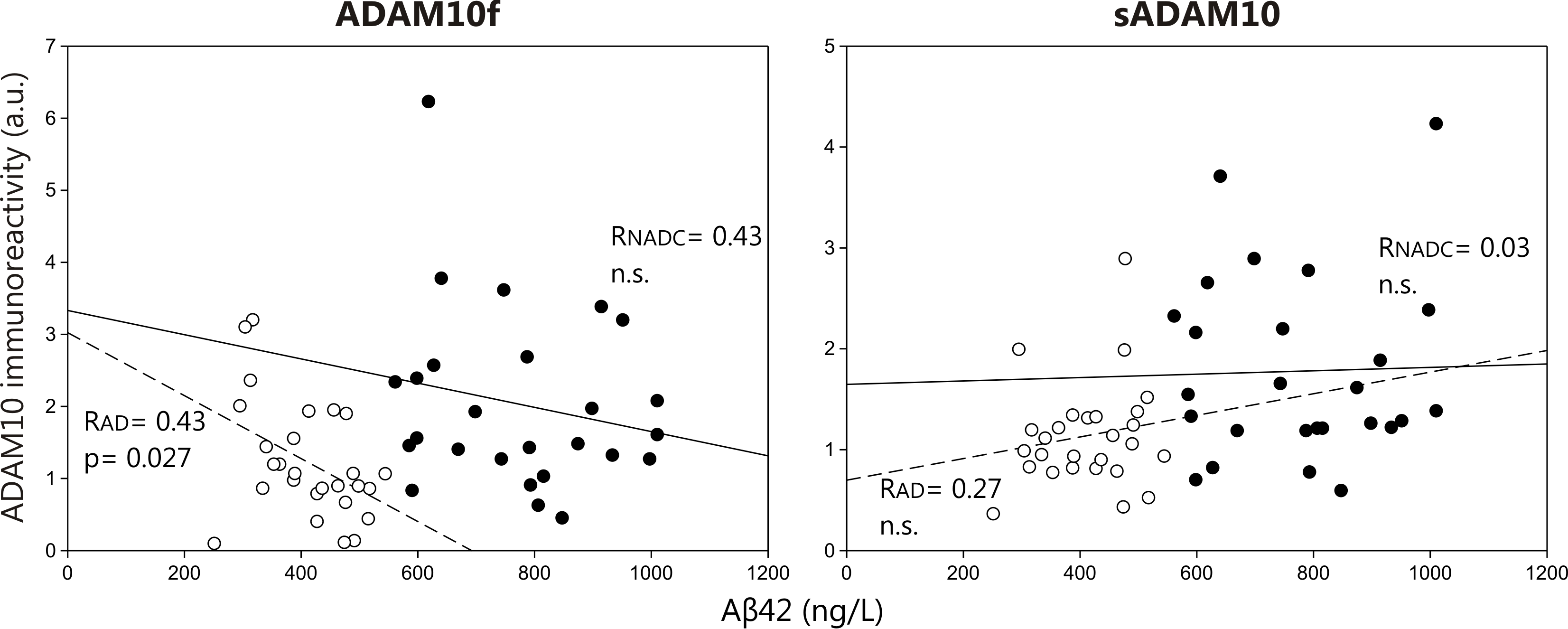

Supplement: Supplementary file 4 — Figure S3. Correlation of mature ADAM10 species with Aβ42 levels in CSF samples. A linear regression analysis was used to assess the correlation between the Aβ42 levels obtained by ELISA (see Table 1) and ADAM10f, or sADAM10 in the samples from age-matched NADC (closed symbol, solid lines) and AD patients (open symbol, dotted lines). Correlations for T-tau or P-tau were non-significant (not shown). The linear regression coefficient (R) and p values for each correlation are shown (n.s.: non-significant p value). (TIF 476 kb) [file 12974_2018_1255_MOESM4_ESM.tif]
